# Work ability and productivity among dentists: associations with musculoskeletal pain, stress, and sleep

**DOI:** 10.1007/s00420-019-01478-5

**Published:** 2019-10-25

**Authors:** Susanna Marklund, Christina S. Mienna, Jens Wahlström, Erling Englund, Birgitta Wiesinger

**Affiliations:** 1 Department of Odontology, Clinical Oral Physiology, Umeå University, 901 87 Umeå, Sweden; 2 Department of Public Health and Clinical Medicine, Occupational and Environmental Medicine, Umeå University, Umeå, Sweden; 3 Department of Research and Development, Umeå University, Sundsvall, Sweden

**Keywords:** Dentist, Pain, Productivity, Sleep, Stress, Work ability

## Abstract

**Purpose:**

Work ability can be measured by the work ability index (WAI), and work-related questions measuring productivity loss in terms of quality and quantity of work. Dentists have high occupational risk of musculoskeletal pain and the exposure of ergonomic strain is already high during dental education. The aim was to evaluate work ability and productivity among dentists, and to identify gender differences and associations with sleep, stress, and reported frequent pain.

**Methods:**

The study population comprised 187 dentists (123 women and 64 men) who had been working as dentists between 5 and 12 years. Participants completed a questionnaire regarding sleep, stress, presence of pain at different sites, work ability assessed by WAI, and productivity in terms of quality and quantity of work.

**Results:**

Poor sleep quality and high level of stress were reported by 31% and 48.1% of participants, respectively, with no gender differences and no association with age. The prevalence of frequent pain ranged 6.4–46.5% with shoulders being the most prevalent site. Thirty-three percent reported reduced work ability. Poor sleep, high amount of stress, and multi-site pain were associated with decreased work ability.

**Conclusions:**

A high prevalence of pain was shown among dentists. Decreased work ability in terms of productivity loss was associated with poor sleep quality, high amount of stress, and multi-site pain. Preventive actions at the workplace should promote good musculoskeletal health, and measures taken, both individual and organizational, to minimize the risk of high, persistent stress and work-related pain.

## Introduction

Work ability has been defined as “the sum of factors enabling an employed person in a certain situation to manage his/her working demands successfully” (Bernburg et al. 2016) or described as the balance between the employee’s/person’s resources and the work demands. Work ability can be measured by the validated work ability index (WAI) (de Zwart et al. 2002). It has also been presented through two work-related questions that measure productivity loss due to pain or discomfort in terms of quality and quantity of work. These questions were presented in 2009 by Martimo et al. (2009), and were recently applied to study associations between neck pain and productivity (Svedmark et al. 2018).

Pain and musculoskeletal disorders (MSD) are common conditions in the general population, and are often linked to sickness absence, long-term sick leave, and decreased quality of life (Breivik et al. 2006; Lindegard et al. 2014). Epidemiological studies have reported that multi-site pain is common in both general and working populations (Carnes et al. 2007; Solidaki et al. 2013). Multi-site musculoskeletal pain was reported to be related to poor work ability (Miranda et al. 2010). In a recent Danish study among female laboratory technicians, work ability decreased gradually with both increased stress levels and the presence of musculoskeletal pain (Jay et al. 2015). High stress levels were also identified as a risk factor for chronic facial pain (Fillingim et al. 2011; Nevalainen et al. 2017) as well as for neck and back pain (Linton 2000). Furthermore, the presence of neck pain was reported to be related to sleep disturbances (Grimby-Ekman and Hagberg 2012; Finan et al. 2013). Recently published data from the Swedish Work Environment Authority show that approximately 1/3 of the working population report neck pain at least once a week (Arbetsmiljöverket 2014).

Among dentists, 25–75% report work-related neck or back pain (Morse et al. 2010), and previous studies have shown the presence of neck and back pain as well as temporomandibular disorders (TMD) already during their dental school period (Thornton et al. 2008; Marklund and Wänman 2010). Dentists are thus at a high occupational risk of contracting musculoskeletal pain in the neck, shoulders, and low back, as well as in the elbows, wrists, and fingers (Morse et al. 2010; Kierklo et al. 2011). Static load on the neck and shoulders, combined with fine motor skills, finger force demands, and time pressure are possible exposures related to the development of pain. In the past, dentistry has been a male-dominated profession, but today, women dominate the occupation. In Sweden, 60–70% of those attending the dental program are women. Previous studies also indicate that MSD are especially common among women working within human services (Leijon et al. 2004). Women in the general population report neck pain more frequently and also seek care to a greater extent than men (Fejer et al. 2006; Larsson et al. 2007). Since women are more likely to develop pain, there is increasing need for continued research on the topic of gender differences and work ability in dentistry.

Several studies show a comorbidity of pain in the jaw, neck, shoulders, and low back (Von Korff et al. 2005; Wiesinger et al. 2007; Wiesinger et al. 2009; Marklund et al. 2010; Graff-Radford 2012), and a mutual influence between these pain conditions has been shown (Marklund et al. 2010). However, there is still a gap of knowledge concerning the relationship between different pain conditions, high amount of stress, and estimated sleep quality, and the impact these factors have on work ability and productivity among dentists. The present study aims to evaluate the work ability and productivity in terms of quality of work and quantity of work among dentists, and to identify gender differences and associations with sleep, stress, and reported frequent pain. The hypothesis was that pain, high amount of stress, and poor sleep were associated with reduced work ability and productivity.

## Subjects and methods

### Study population

A total of 371 individuals who attended the dentistry program at Umeå University during 1998–2005 were invited to participate in this cohort study. They all received a questionnaire, sent by post, during the beginning of 2015, a letter of correspondence, and a reply envelope. After two reminders, a total of 212 responses were received. The dropouts (*n* = 159) did not reply for the following unknown reasons: interrupted dental studies, had an unknown address, answered that they did not want to participate, had emigrated or had died. Those who were working in other professions, were on sick leave, or were on parental leave were excluded from the analyses (*n* = 25). Hence, a study population of 187 working dentists (123 women and 64 men) was identified, all who had been working as dentists between 5 and 12 years. The mean age was 37.4 years (range 31–59 years).

### Ethical considerations

The study was approved by the Regional Ethical Review Board in Umeå, Sweden, (Dnr 2015-86-32M) and carried out in accordance with the Declaration of Helsinki.

### Questionnaire

All participants completed a questionnaire regarding:

- Age
- Gender
- Employment (employee or employer/boss)
- Working hours (full time or part time)
- Presence of frequent pain at different sites—once a week or more often (yes or no)
- Intensity of pain at different sites—jaw/face/temples, head, neck, shoulders, low back, elbows, and wrists/fingers (rating on the Numerical Rating Scale: NRS 0–10 from 0 (practically nothing) to 10 (worst thinkable) (Von Korff et al. 1992))
- General health (rating on a 5-point scale: very good, good, reasonable, poor, or very poor)
- BMI [body mass index (kg/m^2^)]
- Sleep quality (rating on a 5-point scale: very good, good, reasonable, poor, or very poor)
- Amount of stress (rating on a 5-point grading scale: not at all, just a little, to some extent, quite a lot, or very much (Elo et al. 2003))
- Work productivity
  1. Quality of work (rating on the Numerical Rating Scale, NRS 0–10)
  2. Quantity of work (rating on the Numerical Rating Scale, NRS 0–10)
- Questions included in the validated work ability index (WAI) (de Zwart et al. 2002)

Work productivity was assessed with two questions (Martimo et al. 2009):

1. Assess the impact of your symptoms last month and mark on a scale from 0 (very poor quality) to 10 (regular quality) the quality of your work when compared to your normal workday;
2. Assess the impact of your symptoms last month and mark on a scale from 0 (practically nothing) to 10 (regular quantity) how much work you were able to perform when compared to your normal workday.

### Analysis

For the analyses, the items below were dichotomized as follows:

- Reported pain: no pain (less than once a week) or frequent pain (once a week or more)
- Reported sleep quality: good (very good, good) or poor (reasonable, poor, very poor)
- Reported level of stress: low (not at all, just a little) or high (to some extent, quite a lot, very much)
- Quality of work: high (NRS 9–10) or reduced (NRS 0–8)
- Quantity of work: high (NRS 9–10) or reduced (NRS 0–8)

The calculation of the WAI score was based on the answers to seven in-depth questions about physical and mental demands of the work, as well as the worker´s health state and mental resources. Based on the individual total score, each person was classified and grouped into one of four WAI categories: poor (7–27 points); moderate (28–36 points); good (37–43 points); and excellent work ability (44–49 points) (de Zwart et al. 2002).

The variable number of pain sites was created with categories 0 (no frequent pain), 1 (one site with frequent pain), and > 1 (more than one site with frequent pain). Pain in the elbows and wrists/hands/fingers was pooled.

### Statistics

The Chi-square test was used to test for associations between gender and reported sleep quality, level of stress, WAI, and occurrence of frequent pain at different sites. The Mann–Whitney *U* test was used to test for associations between age and sleep, stress, quality of work, quantity of work, as well as the number of pain sites, respectively. Logistic regression analyses were performed with quality of work and quantity of work as output variables. For each of these variables, the factors gender, estimated sleep quality, amount of stress, and number of pain sites were entered as covariates. The logistic regression was first calculated for each factor and each dependent variable, adjusted only for gender. Then, a multivariate model was created using backward selection method, run in a manual procedure, and adjusted for gender. In the first step, all four factors (gender, estimated sleep quality, amount of stress, and number of pain sites) and all second-order interactions were entered. Step by step, the non-significant interactions were removed, followed by removal of non-significant single factors, except for those associated with significant interactions. WAI was not included in the logistic regression analyses due to few individuals in the poor and moderate categories.

For quality of work and quantity of work, the interaction between stress and number of pain sites was not entered into the model because of zero cells. The same applied to the interactions between sleep and number of pain sites for quantity of work.

Associations between output variables and covariates were assessed by odds ratios (OR) and 95% confidence intervals (CI). In the regression analysis, the results were considered statistically significant if the CI did not include 1 (one). A *p* value < 0.05 was considered statistically significant. All analyses were performed with IBM SPSS 24 (IBM Corp, Armonk, NY).

### Quality control and assurance

Data were collected during a limited period of time and registered in excel and SPSS by one person. Before the analyses, data were checked twice. The analyses were performed with well-tested statistical tools and techniques, by an experienced statistician. All authors are well acquainted with the data and the preparation of the manuscripts.

## Results

### Population characteristics

Population characteristics are presented in Table 1. Poor sleep quality and high level of stress were reported by 31% and 48.1%, respectively, with no gender differences and no association with age. WAI was poor for 0.6% of the participants, whereas 5.8% had moderate, 34.9% good, and 58.7% had excellent work ability, respectively, without gender differences. Reduced quality of work and reduced quantity of work were reported by 19.8% and 20.2%, respectively, of those included in the analysis. There were no associations between age and work productivity.

**Table 1:** Population characteristics (*n* = 187)

	Number of participants	%	Mean	SD
Gender				
Male	64	34.2		
Female	123	65.8		
BMI				
Range 18–39	185	98.9	23.7	3.4
Missing	2	1.1		
Age				
Range 31–59	187	100	37.4	5.1
General health^a^				
0	175	94.1		
1	11	5.9		
Missing	1			
Employment				
Employee	141	75.4		
Employer/boss	46	24.6		
Working hours				
Full time	118	63.1		
Part time	69	36.9		
Sleep^a^				
0	129	69.0		
1	58	31.0		
Stress^b^				
0	97	51.9		
1	90	48.1		
Quality of work^c^				
0	138	80.2		
1	34	19.8		
Missing	15			
Quantity of work^c^				
0	138	79.8		
1	35	20.2		
Missing	14			
Number of pain sites				
0	54	28.9		
1	33	17.6		
> 1	100	53.5		
WAI				
Range 23–49			43.4	4.5
Poor	1	0.6		
Moderate	10	5.8		
Good	60	34.9		
Excellent	101	58.7		
Missing	15			

*BMI* body mass index, *WAI* work ability index (WAI score: poor = 7–27, moderate = 28–36, good = 37–43, excellent = 44–49)

^a^0 = very good, or good; 1 = reasonable, poor, or very poor

^b^0 = not at all, or just a little; 1 = to some extent, quite a lot, very much

^c^0 = high (NRS 9–10), 1 = reduced (NRS 0–8)

### Musculoskeletal symptoms

**Fig. 1:**
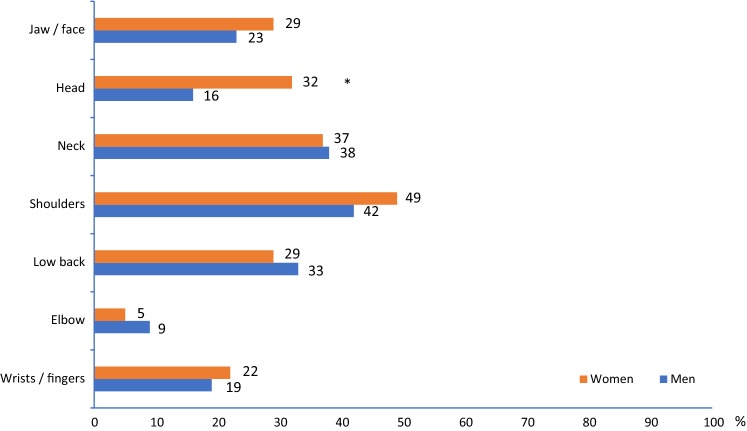
Location and gender distribution of reported frequent pain (%). *p = 0.02

**Fig. 2:**
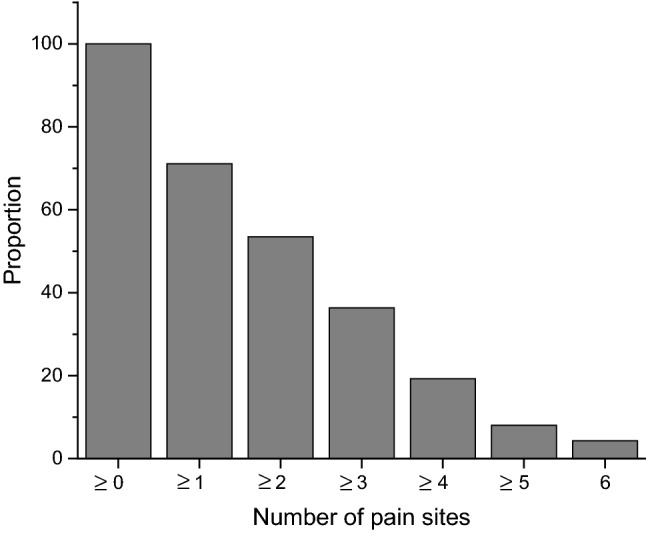
Reverse cumulative proportions of the number of pain sites

In total, 71.1% (men 64.1%, women 74.8%) of the subjects reported frequent pain (once a week or more) in the jaw/face/temples, head, neck, shoulders, low back, elbow, or wrist/hand/fingers. Frequent pain at these sites was reported within the range 6.4–46.5%. Location and gender distribution are presented in Fig. 1. Shoulders were the most common pain site among both men (42.2%) and women (48.8%). No gender differences were found for the prevalence of pain at different sites, except for frequent headaches (15.6% among men and 31.7% among women, *p* = 0.02). The reverse cumulative proportion of the number of pain sites is shown in Fig. 2. There was no association between age and number of pain sites.

### Work productivity in terms of work quality and quantity in regression models

In any of the logistic regression models, gender itself was not significant (quality of work: OR 1.3; 95% CI 0.6–2.9) and quantity of work: OR 1.0; 95% CI 0.4–2.1). In the multivariate regression analysis, reduced quality of work showed significant associations with high level of stress (OR 5.0; 95% CI 2.0–12.3) and presence of more than one pain site (OR 7.2; 95% CI 1.6–32.0) (Table 2). Reduced quantity of work showed significant associations with estimated poor sleep (OR 3.5; 95% CI 1.6–7.5), high level of stress (OR 4.3; 95% CI 1.8–10.0), and presence of at least one pain site (OR 17.0; 95% CI 2.2–129.4) (Table 2).

**Table 2:** Binary logistic regression analysis of quality of work (high or reduced) and quantity of work (high or reduced) as outcome variables and symptoms as covariates

	Quality of work^c^			Quantity of work^c^		
	Number (*n* = 172)	Adjusted^#^ OR (95% CI)	Multivariate OR (95% CI)	Number (*n* = 173)	Adjusted^#^ OR (95% CI)	Multivariate OR (95% CI)
Gender						
Male	58	1.0	1.0	58	1.0	1.0
Female	114	1.3 (0.6–2.9)	1.0 (0.4–2.3)	115	1.0 (0.4–2.1)	0.6 (0.3–1.5)
Sleep^a^						
0	118	1.0		119	1.0	1.0
1	54	2.3 (1.1–5.1)*		54	3.5 (1.6–7.5)**	2.9 (1.3–6.8)*
Stress^b^						
0	85	1.0	1.0	85	1.0	1.0
1	87	5.0 (2.0–12.3)***	4.3 (1.7–10.8)**	88	4.3 (1.8–10.0)***	3.3 (1.3–8.4)*
Number of pain sites						
0	42	1.0	1.0	42	1.0	1.0
1	32	4.6 (0.9–24.6)	4.7 (0.8–26.4)	32	7.6 (0.8–68.6)	12.8 (1.3–130.3)*
> 1	98	7.2 (1.6–32.0)**	5.3 (1.2–24.6)*	99	17.0 (2.2–129.4)**	16.4 (2.0–133.7)**

OR (odds ratio) and CI (confidence interval) are reported

*p < 0.05, **p < 0.01, ***p < 0.001

^#^Adjusted for gender

^a^0 = very good, or good; 1 = reasonable, poor, or very poor

^b^0 = not at all, or just a little; 1 = to some extent, quite a lot, very much

^c^High = NRS 9–10, reduced = NRS 0–8

## Discussion

The main findings of the present study were that approximately 20% of the dentists reported decreased productivity in terms of quality and quantity of their work due to pain or discomfort, and just over 6% reported poor-to-moderate work ability, according to work ability index (WAI). Poor sleep quality, high level of stress, and multi-site pain were also common and were all associated with productivity in the analyses adjusted only for gender. Consequently, the hypothesis was met. In the multivariate model, poor sleep did not remain significant for decreased quality of work.

For the definition of work ability, we used the validated work ability index (WAI) (de Zwart et al. 2002), and also the two questions measuring self-rated productivity in terms of quality of work and quantity of work (Martimo et al. 2009). In the study by Martimo et al. (2009), the self-reported productivity loss was 56% among active workers in various physical, as well as sedentary, occupations who were clinically diagnosed with an upper extremity musculoskeletal disorder. In the present study, we found a lower value with approximately 20% reporting reduced quality of work or quantity of work, respectively. On the other hand, our definition of productivity loss was 0–8 on an NRS 0–10, whereas Martimo regarded 0–9 as productivity loss. In the present study, cut-off was chosen to get enough individuals (*n*) in each group for the analyses.

Having a clinical diagnosis, which is most common with reported multi-site pain, could thus imply a more severe productivity loss than having symptoms without diagnosis. We do not know if our subjects had any clinical diagnosis, but they did have symptoms of multi-site pain, which probably means that some also had clinical diagnosis. In support of this is our data with the high odds ratios for decreased productivity among dentists with more than one pain site. This is also in line with a recent study among health care providers, including dentists, that showed that multi-site musculoskeletal pain was associated with poor work ability and that the association was likely to increase by a higher number of pain sites (Phongamwong and Deema 2015).

In a systematic review, poor work ability was associated with reduced musculoskeletal capacity, lack of physical activity on leisure time, high physical and psychosocial work demands, older age, and obesity (van den Berg et al. 2009). Psychosocial work environment factors such as job strain, control, demands, and perceived stress were also reported to be associated with decreased productivity and work ability (Martimo et al. 2009). Furthermore, a recent study by Svedmark et al. (2018) showed associations between stress and decreased productivity. People with reduced working capacity more often report high work-related stress. An elevated risk of impaired work ability and chronic MSD was found among individuals in professions; for example, dentists, who are exposed to repetitive, monotonous exertion, and body positions (Jay et al. 2015). The work situation for dentists involves high clinical job skills combined with cost–effectivity demands and time pressure. Working under high workloads will affect the dentist´s stress levels and work ability in the long run. In the present study, we found strong associations between multi-site pain, high amount of stress, and reduced work productivity. Among female laboratory technicians, increased self-perceived stress and musculoskeletal pain from the neck and shoulders were independently associated with lower work ability (Jay et al. 2015). In addition, previous studies showed associations between temporomandibular disorders (TMD) and stress (De Leeuw et al. 2005; Sipila et al. 2008; Fillingim et al. 2011). In a recent study from Finland among the general population, high stress levels were connected to an increased risk for chronic facial pain in comparison to low stress levels as assessed by items in the WAI (Nevalainen et al. 2017).

Lindegård et al. (2014) reported that for Swedish health care workers, high job strain, sleep disturbances, and musculoskeletal pain were associated with decreased work performance and productivity loss. The concept of work performance was defined as a combination of qualitative and quantitative aspects of performing a work task. Frequent musculoskeletal pain was identified as a risk factor for decreased work ability and work performance.

We found high frequencies for pain in different sites with the highest prevalence for the neck and shoulders. The values were near to or slightly lower than those reported by others (Morse et al. 2010; Sakzewski and Naser-ud-Din 2014; Pejcic et al. 2017; Taib et al. 2017). In different studies, different measurement tools have been used to determine MSDs. In our study, reported pain once a week or more often was included in the analysis. In several similar studies, a modification of the Standardized Nordic Questionnaire was used, then as reported pain or discomfort during work for the last 12 months (Kuorinka et al. 1987), without taking into account the frequency and intensity of the symptoms. Using different measurement tools or different definitions regarding the presence of musculoskeletal pain symptoms may account for the different prevalence values, and thus may complicate comparisons between studies.

To our knowledge, the present study is the first where work ability and productivity were calculated among dentists and that work productivity related to stress, sleep quality, and reported frequent pain are examined among dentists. Data were collected with the aid of a questionnaire. The response rate was 57%. This was acceptable and in line with some corresponding studies (Sakzewski and Naser-ud-Din 2015; Pejcic et al. 2017), but slightly lower than that reported in a recent review by Morse et al. (2010). Among those who did not respond, the distribution between men (46%, *n* = 73), and women (54%, *n* = 86) were more equal compared to those who responded. The age range was between 18 and 42 years, and the majority (80% of those who did not respond) were 27 years or younger.

One of the strengths of our study was the use of validated screening questions, for example, validated screening questions for calculating the WAI score. Work ability was assessed by the work ability index (WAI), which is an internationally well-accepted and validated questionnaire tool developed by the Finnish Institute of occupational health (Ilmarinen 2007). The use of such questions is a valid and applicative tool in occupational health research as well as in the daily practice of occupational health care. On the other hand, a major limitation of our study is the cross-sectional design which limits conclusions regarding causality. Another limitation is that the questionnaire survey may compose a retrospective bias. On the other hand, this applies to all participants in the study.

Based on the information which we have about working hours, no conclusions can be drawn regarding possible associations to any of the studied variables. About 1/3 of the respondents work part time, but the majority, 155 individuals (83%) work more than 80% up to full time. The reason for working part time is not known, but 97% of these individuals are 32–41 years, which reflects a time in the middle of life, with probable periods of parental leave. When it comes to type of employment, one can suspect that the employer/boss has greater influence over work compared to the employee. However, we did not find any associations between employment type and work productivity, sleep quality, amount of stress, or various pain symptoms.

It is remarkable that every fifth dentist rather early in their careers report reduced quality of work due to pain or discomfort. This could affect the care received by the patients. In this study, it becomes clear that many working dentists consider themselves healthy and capable of work, despite having frequent pain. Here, the WAI score does not capture these problems while the calculations on work productivity do. The relationship between the dentist’s work environment and their own health is now an identified area that needs further exploration.

## Conclusion

About 20% of the dentists reported decreased productivity of their work due to pain and discomfort, and over 6% reported poor-to-moderate work ability. Productivity loss was associated with poor sleep quality, a high amount of stress, and multi-site pain. Preventive actions at the workplace to maintain high work ability and productivity should include measures to promote good musculoskeletal health among dentists, as well as measures, both individual and organizational, to minimize the risk of high, persistent stress levels, and frequent work-related pain. These symptoms must be identified early and taken seriously as pain is a strong predictor for persistent stress and future inconvenience.
